# Frequency of wrist pain and its associated risk factors in students using mobile phones

**DOI:** 10.12669/pjms.36.4.1797

**Published:** 2020

**Authors:** Fatima Amjad, Muhammad Nazim Farooq, Rubia Batool, Anam Irshad

**Affiliations:** 1 Fatima Amjad, DPT. Islamabad College of Physiotherapy, Margalla Institute of Health Sciences, Rawalpindi, Pakistan; 2 Muhammad Nazim Farooq, PhD. Islamabad College of Physiotherapy, Margalla Institute of Health Sciences, Rawalpindi, Pakistan; 3 Rubia Batool, DPT. Fauji Foundation Hospital, Rawalpindi, Pakistan; 4 Anam Irshad, DPT. Benazir Bhutto Hospital, Rawalpindi, Pakistan

**Keywords:** Cell phone, Pain, Frequency, Risk factors, Students, Wrist

## Abstract

**Objective:**

To assess the frequency of wrist pain in students due to mobile phone usage, and impact of usage hours and screen size of mobile phones on pain and disability at wrist joint.

**Methods:**

A cross-sectional survey was conducted among students studying in different universities of Islamabad and Rawalpindi belonging to both public and private sectors. The study was conducted between May 2018 and March 2019. Sample size was 360 students which were selected through convenience sampling. Data was collected through self-formulated closed ended questionnaire. Patient Rated Wrist Evaluation questionnaire was used to assess pain and disability at wrist joint. Data entry and analysis were done using SPSS 21. Results were analyzed using descriptive statistics. Spearman’s and point-biserial correlation coefficients were applied to determine association between different variables.

**Results:**

Point, last month, last 3 months, last 6 months, last year and lifetime frequency were found to be 9%, 18.6%, 29%, 33.3%, 42% and 45.3% respectively. Duration of mobile phone usage was found to be of significant association factor that could lead to wrist pain and disability (p=0.004). Wrist pain was not significantly related to mobile phone screen size (p=0.488).

**Conclusion:**

It appears that wrist pain is common among mobile phone users and an increase in use of mobile phones increased pain and disability of wrist joint. In addition, it seems that screen size of mobile phone has no significant effect on pain and disability of wrist joint.

## INTRODUCTION

Mobile phones are omnipresent in the 21^st^ century. This little device has changed the way people communicate locally and globally. The desire and urge to stay connected and online round the clock have turned into a basic need in the modern day interactive communications and cell phone has proved to be the most convenient and accessible tool of choice. Too much of a good thing can usually have some downsides, and texting on your favourite smartphone is no exception. This extreme level of indulgence and dependence on technology often makes us oblivion to its harms in the long run. It is an indispensable item when on the go. The purpose of use is chatting, tweeting, social media interactions, documents formatting and other activities.

People who spend majority of time on their smartphones may be scrolling, tapping and swiping their way to a painful wrist and hand disorder.^1^ Musculoskeletal disorders are caused by sudden exertion or prolonged exposure to physical factors such as prolonged, forceful, low amplitude, repetitive use of such devices.^2^ Mahaba et al.^3^ investigated probable subjective hazards of mobile phones in a study that stated wrist is significantly affected by use of mobile phones. It included 692 students and employees from university and concluded that wrist pain was felt by 438 (63.3%) participants. A study in Ziauddin College of Physiotherapy, Karachi Pakistan concluded that there does exist an association between wrist/thumb pain due to excessive use of phones for texting.^4^ In this study, 125 (42%) students exhibited pain like symptom in wrists and thumbs.^4^ Sharan et al.^2^ found that among 27 subjects who participated in this study 14.81% of the participants were diagnosed with wrist tendinitis. Berolo et al.^5^ investigated musculoskeletal symptoms among mobile users in a Canadian university and found 137 of 140 participants (98%) reported pain. Right hand pain was most common among users. Woo et al.^6^ performed a study on 503 university students, aged 18-25 years, in the university of Hong Kong and found that half of the respondents (43.4%) reported discomfort wrist/hand areas.

Although the importance of studying wrist joint hazards by mobile phones can’t be overlooked because of its extensive worldwide use, relatively scarce studies were conducted to study its possible hazards to the wrist joint. Most of investigators were interested in determining the possible neck and upper extremity symptoms and the other work done earlier was to find the cumulative effects on the wrist joint which mainly can cause De Quervain’s tenosynovitis and carpal tunnel syndrome due to frequent text messages. However, less attention was given more solely to the wrist joint.

The main focus of this study was to assess the frequency of wrist pain in students due to mobile phone usage and determine the impact of usage hours and screen size of mobile phones on pain and disability at wrist joint.

## METHODS

A cross-sectional survey was conducted among students studying in universities of Islamabad and Rawalpindi. The study was conducted between May 2018 and March 2019. Sample size was 360. Sample was selected through non probability convenience sampling technique. Students with 18 to 25 years of age using touch screen mobile phone for any duration were included in the study. Students with recent accident or trauma of wrist joint, recent history of taking analgesics, liver disease, carpal tunnel syndrome, neurological deficit, inflammatory arthropathy, history of fracture of wrist joint and diabetes were excluded.

Data collection was done by using self-structured questionnaire. The questions were generally close ended, including information such as type of mobile phone used by the students, number of hours spent on mobile phone per day, screen size of mobile phone, experiencing pain in wrist joint and any treatment received for wrist pain. In addition to questionnaire, pain and disability was assessed through Patient Rated Wrist Evaluation questionnaire (PRWE).^7^ PRWE is a 15-item questionnaire designed to measure pain and disability at wrist joint. It allows patients to rate their wrist pain and disability levels from 0 to 10, and consists of 2 subscales: pain subscale and function subscale. Pain subscale contains 5 items. Function subscale is further divided into 2 sections i.e. specific activities (having 6 items) and usual activities (having 4 items). The maximum score of each subscale is 50 and minimum is 0. Test-retest reliability of PRWE was found excellent (ICCs > 0.90).^7^ Validity assessment showed significant differences in it over time (p < 0.01).^7^

### Ethical approval

Ethical approval was obtained from the ethical review committee of Margalla Institute of Health Sciences. Rawalpindi (Ref No. RB/43/18, dated May 14, 2018). Participants were provided with right to ask any question about study and they were free to refuse any part of study without affecting their relationship with investigator. A written consent form was signed by each student who participated in study.

### Sample size

Sample size was calculated using the formula for epidemiological studies. Using frequency rate of 63% reported by the earlier study,^3^ 95% confidence interval and 5% error of frequency rate 359 participants were required which were rounded to 360.

### Data analysis

Data entry and analysis were done using computer software SPSS version 21. Frequency and percentages were taken for the continuous and categorical variables. Spearman’s and point-biserial correlation coefficients were applied to determine association between different variables. The level of statistical significance was set at 0.05.

## RESULTS

Total 449 questionnaires were distributed to students. Out of which, 423 questionnaires were filled and returned. An aggregate of 63 questionnaires were excluded because they did not fulfill the inclusion criteria. Out of the data obtained, there was female predominance of 64% (n = 231) while remainder 36% (n=129) of study population included male students. Sample characteristics and frequency of wrist pain at different periods of time are shown in Tables I and II respectively.

**Table-I:** Sample Characteristics.

Variables	Mean	Standard deviation
Age (years)	21.07	± 1.83
Height (inches)	64.99	± 3.62
Weight (lbs.)	126.70	± 22.82
Body mass index	21.07	± 3.31

**Table II:** Frequency of Wrist pain.

Period of Frequency	Frequency rate %
Point Frequency	9%
Last Month Frequency	18.6%
3 Months Frequency	29%
6 Months Frequency	33.3%
Annual Frequency	42%
Lifetime Frequency	45.3%

The mean and standard deviation of the wrist pain and disability on PRWE were 11.58 ± 14.02. The frequency and percentages of the severity of wrist pain and disability in the students are summarized in Table-III. No student was found to have very severe (81-100) wrist pain and disability.

**Table-III:** Wrist pain and disability.

Wrist pain and disability	Frequency	Percent
None (0)	108	30%
Minimal (1-20)	172	48%
Mild (21-40)	62	17.2%
Moderate (41-60)	15	4.2%
Severe (61-80)	3	0.8%

The time spent by students on mobile phones is shown in Table-IV. A significant positive correlation was found between mobile phone usage hours and wrist pain and disability (r=0.223, p<0.001). It showed that as duration of mobile phone usage by students increases the severity of their wrist pain and disability increases as well.

**Table-IV:** Mobile Phone Usage Hours.

Duration	Frequency	Percent
0-1 hour	28	7.8
2-3 hours	85	23.6
3-5 hours	94	26.1
More than 5 hours	153	42.5

It was found that 70% of the students have mobile phones with screen size of greater than 5 inches. The remaining 30% of the students have mobile phones with screen size of less than 5 inches. A non-significant association was found between screen size and wrist pain and disability (r_pb_=-0.024, p=0.647).

## DISCUSSION

The present study found that wrist pain was a common complaint among students due to mobile phone usage. The point frequency came out to be 9% which was consistent with findings by Sharan et al.^2^ However, it was a little higher than the findings of Stalin et al.^8^ This might be due to the difference in area of pain reported. For example, they reported the frequency of pain in fingers rather wrist due to mobile phone usage. Furthermore, the current results of annual frequency (42%) are consistent with the findings of previous work.^6^

The lifetime frequency was found comparable to a previous study by Ali et al.^4^ However, this was contrary to the results of a study by Parasuraman et al.^9^, who reported 26% frequency of wrist pain. This difference may be due to variance in duration of mobile phone usage per day. For example, in a study by Parasuraman et al.^9^, 64% respondents were using mobile phones for roughly less than one hour, whereas in the current study the majority of the participants have mobile phone usage of more than five hours per day. The findings of the current study were also varying with the results of Kim et al.^10^ who reported wrist pain in 27.1% of students. However, in this study the frequency period was unclear. On the other hand, Mahaba et al.^3^ reported high frequency of wrist pain (63.3%) compared with the findings of current study. It might be for the reason that they have reported the cumulative frequency of pain in fingers, hand and wrist.

The current study found a significant association between spending long hours on smartphones and increase in wrist pain and disability. This is in agreement with the findings of the earlier studies which supports the notion that increasing the usage hours increases the pain and disability in wrist joint.^5^,^11^-^13^ It appeared that long duration of mobile phones usage can cause vulnerability to wrist joint. The repetitive movements and different positions of the wrist joint while using mobile phone can cause effect on the structures of the wrist joint.^4^,^6^ In the current study, 42% students were found to spend more than 5 hours on the mobile phone which is steady with the work done formerly.^6^,^14^

A non-significant association was found between screen size of mobile phone and pain and disability of wrist joint. This was supported by the previous work about the association of screen size and impact on wrist pain.^10^ It appears that screen size of mobile phone does not have a significant impact on pain and disability at wrist joint.

PRWE was used to assess the pain and disability of wrist joint. The results showed that a large number of students using mobile phones were affected by pain and disability at wrist. This is in agreement with the findings of the studies done by Berolo et al.^5^ and Balakrishnan et al.^15^

### Limitations of the study

The study may be limited due to cross sectional study design, changes over time cannot be determined. The small sample size of this study and age criteria (18-25) may not have been sufficient enough to generalize the result of our findings over a larger population.

## CONCLUSION

On the basis of findings several conclusions concerning effects of mobile phone usage on wrist joint can be drawn. The findings of this study indicated that frequency of wrist pain amongst mobile phone users appears to be high. The duration of mobile phone usage had a significant association with pain and disability of wrist joint. Association of mobile phone’s screen size with pain and disability of wrist joint was not found significant.

### Authors’ Contribution

**FA, RB and AI:** Concept, design, literature review, data collection, data analysis, writing of manuscript.

**MNF:** Concept, design, literature review, analysis and interpretation of data, critical revision of the article for important intellectual content. Responsible and accountable for the accuracy or integrity of the work.

All authors have read and approved the manuscript.
